# Investigation on the Interaction between Cyclophosphamide and Lysozyme in the Presence of Three Different Kind of Cyclodextrins: Determination of the Binding Mechanism by Spectroscopic and Molecular Modeling Techniques

**DOI:** 10.3390/molecules18010789

**Published:** 2013-01-11

**Authors:** Mahdieh Mansouri, Malihe Pirouzi, Mohammad Reza Saberi, Maryam Ghaderabad, Jamshidkhan Chamani

**Affiliations:** 1 Department of Biology, Faculty of Sciences, Mashhad Branch, Islamic Azad University, Mashhad 9175687119, Iran; E-Mails: mansori.mh@gmail.com (M.M.); pirozi8@gmail.com (M.P.); ghader.abad.m@gmail.com (M.G.); 2 Medical Chemistry Department, School of Pharmacy, Mashhad University of Medical Science, Mashhad 9175687119, Iran; E-Mail: saberimr@mums.ac.ir

**Keywords:** lysozyme, cyclophosphamide, CDs (α,β,γ), fluorescence quenching, circular dichroism, molecular modeling

## Abstract

The interactions between cyclophosphamide (CYC) and lysozyme (LYZ) in the presence of different cyclodextrins (CDs) were investigated by UV absorption, fluorescence spectroscopy, circular dichroism (CD), and molecular modeling techniques under imitated physiological conditions. The UV absorption results showed the formation of complexes between CYC and LYZ in the presence of different CDs. Fluorescence data show that CYC has a stronger quenching effect on LYZ, and the red shifts suggested that the microenvironment of Trp residues was changed and became more hydrophilic. The interaction of CYC with LYZ and quenching properties of the complexes caused strong static fluorescence quenching in binary and ternary systems. The binding affinities as well as the number of binding sites were obtained from interaction between CYC and LYZ in the presence of different CDs as binary and ternary systems by modified Stern-Volmer plots. The Resonance Light Scattering (RLS) technique was utilized to investigate the effect of drug and CDs on conformational changes of LYZ as separate and simultaneous. The results suggested that the enhancement of RLS intensity was attributed to the formation of a complex between drug and protein in absence and presence of CDs. The effect of CYC and cyclodextrins on the conformation of LYZ was analyzed using synchronous fluorescence spectroscopy. Our results revealed that the fluorescence quenching of LYZ originated from the Trp and Tyr residues, and demonstrated conformational changes of LYZ with the addition of CYC and CDs. The molecular distances between the donor (LYZ) and acceptor (CYC and CDs) in binary and ternary systems were estimated according to Forster’s theory and showed static quenching for protein with CYC in the presence of CDs. The CD spectra indicated that the binding of the CYC induced secondary structural changes in LYZ in binary and ternary systems. Molecular modeling suggested the binding sites of CYC in the ternary systems differ from those in the binary systems. estimated the distance between CYC and Trp residues in binary and ternary systems in the presence of CDs and confirmed the experimental results.

## 1. Introduction

Egg white constitutes an attractive source containing many useful proteins. Lysozyme (LYZ), one of the proteins present in egg white muramidase or *N*-acetylmuramide glycanohydrolase, is abundant in a number of secretions, such as tears, saliva, human milk and mucus. Lysozyme can attack peptidoglycans (found in the cell walls of bacteria, especially Gram positive bacteria) and hydrolyze the glycosidic bond that connects N-acetylmuramic acid with the fourth carbon atom of *N*-acetyl glucosamine [1].

It is also a small monomeric globular protein, consisting of 129 amino acid residues and containing six Trp and three Tyr residues. Three of the Trp residues are located at the substrate binding sites, two in the hydrophobic matrix box, while one is separated from the others. LYZ has many physiological and pharmaceutical functions [2], one of which is the ability to carry drugs and the effectiveness of drugs depends on their binding ability. Therefore, studies on the interaction between drugs and LYZ are of importance in view of understanding the disposition, transportation and metabolism of drugs as well as the efficacy of processes involving drug and LYZ [3].

**Figure 1:**
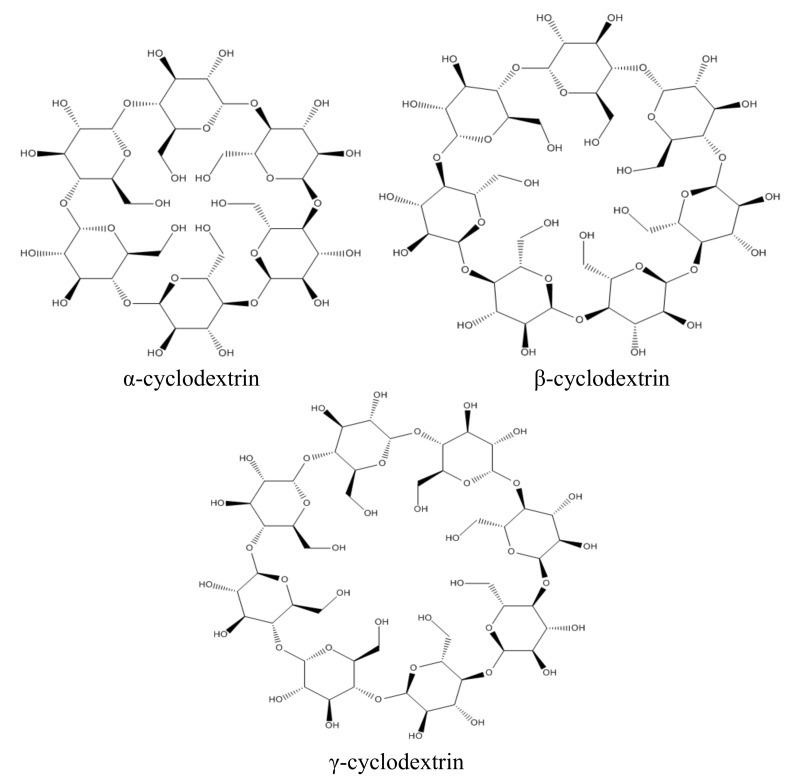
The chemical structures of cyclodextrins (α,β,γ).

Native CDs (Figure 1) are among the most interesting and functional host natural materials having a rigid, well defined ring structure, with the shape of a truncated cone, and binding ability in their hydrophobic cavity via hydrophobic, van der Waals and hydrogen bond interactions [4,5]. There exist three stable forms of CD: α, β and γ, differing in the quantity of glucose molecules in the ring (from six to eight) and the corresponding diameter of the ring [6]. All of the OH groups in CDs are on the outer surface of the molecule, therefore the inner cavity of CDs presents as hydrophobic [7]. CDs are capable of including different molecules into these hydrophobic cavities and forming nanocomplexes transporting the active molecule. These specific characteristics make CDs suitable as aqueous solubilizers of various kind of lipophilic chemicals, for example drugs. CDs are widely used in the food, pharmaceutical, medical, chemical, and textile industries [8].

**Figure 2:**
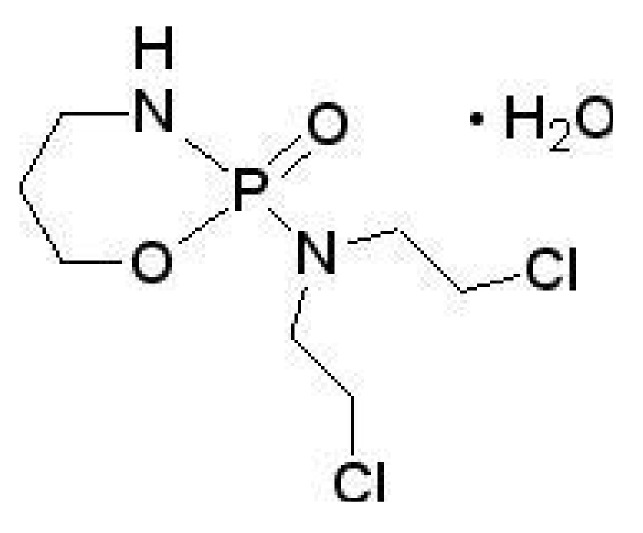
The chemical structure of cyclophosphamide.

Cyclophosphamide (CYC, Figure 2) is a white crystalline powder with a molecular weight of 279 Da. CYC is soluble in water, saline solution or ethanol, and drug interactions with proteins will in most cases significantly affect its elimination rate. Cyclophosphamide is used as an anticancer drug, employed in the treatment of metastatic breast cancer. It is an inactive prodrug that requires metabolic activation by the cytochrome P-450 system [9]. Multiple clinical studies have suggested that the use of antioxidants in combination with chemotherapy and irradiation prolongs the survival time of patients compared with the expected outcome without antioxidant supplements [10].

Here, the binding interaction between LYZ with CYC in the prescence of CDs (α,β,γ) was investigated. Attempts were made to investigate the binding mechanism, binding constants, the number of binding sites, determining the binding parameters and transfer efficiency of energy were measured by biophysical methods, mainly resonance light scattering and fluorescence quenching. On the other hand, the conformational changes of LYZ are discussed on the basis of synchronous fluorescence, UV/Vis, and CD spectroscopy data. These studies should be of use in pharmaceutical development, pharmacokinetics and drug delivery.

## 2. Results and Discussion

### 2.1. UV-Vis Absorption Measurements

**Figure 3:**
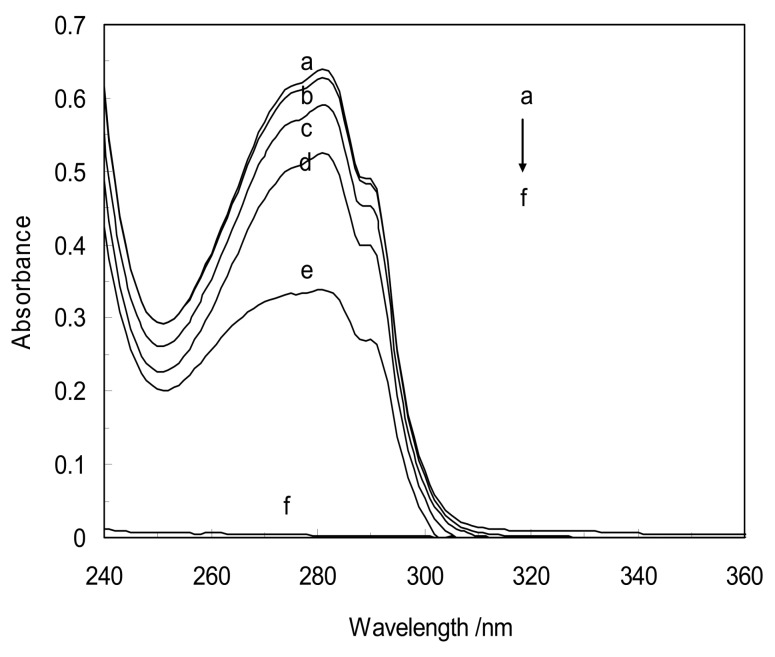
UV/vis absorbance spectra of: **a**: LYZ-CYC; **b**: [LYZ-CD(α)]CYC; **c**: LYZ; **d**: [LYZ-CD(γ)]CYC; **e**: [LYZ-CD(β)]CYC; **f**: CYC, in pH = 7.4 and T = 298 K. [CYC] = 3.5 × 10^−6^ mM and [CDs] = 0.04 mM in all complexes.

Figure 3 shows the absorbance of CYC, LYZ, LYZ-CYC and [LYZ-CD (α,β,γ)]CYC complexes. It can be seen the absorbance of LYZ increases with increasing CYC concentration in binary systems. This indicates that there is an interaction between LYZ and CYC, involving the formation of a ground state complex of the LYZ-CYC type. In the presence of CDs (α,β,γ) in ternary systems, the absorption spectra were different. It can be seen that CD (α) enhances LYZ, and the absorbances increase and decrease gradually with increasing concentrations of CDs β and γ, respectively. The above results suggest that the structural conformation of LYZ has been changed [11].

### 2.2. Fluorescence Quenching of LYZ-CYC Complex in Binary Systems

Most proteins can emit intrinsic fluorescence after absorbing ultra-violet light providing that they comprise residues such as Trp, Tyr and Phe in their molecular structure. The fluorescence intensity of a protein can be weakened by a variety of molecular interactions, which is called fluorescence quenching [12].

The conformational changes of LYZ can be evaluated by measuring the intrinsic fluorescence intensity of LYZ before and after the addition of drugs. Fluorescence measurements also provide information about the molecular environment in the vicinity of chromophore molecules and changes in the emission spectra of Trp often occur in response to conformational transitions, subunit association, substrate binding or denaturation. These interactions can affect the local environment surrounding the indole ring [13].

The absorption of proteins at 280 nm is due to both Tyr and Trp residues, whereas at wavelengths longer that 295 nm, the absorption is primarily due to Trp. Trp fluorescence can thus be selectively excited at 295–305 nm. Tyr is often regarded as a rather simple fluorophore [14].

**Figure 4:**
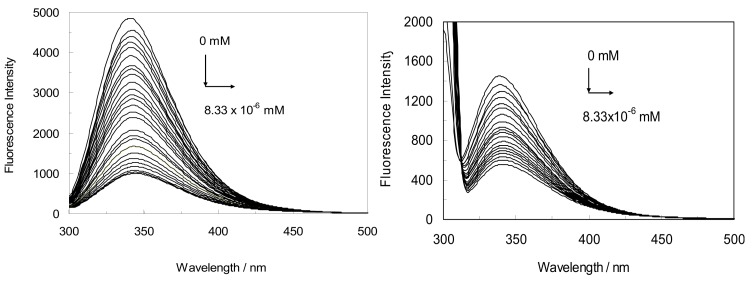
The fluorescence spectra of LYZ-CYC. Conditions: T = 298 K, pH = 7.4, λ_ex_ = 280 nm. (right; λ_ex_ = 295 nm), The concentration of LYZ was 1 × 10^−3^ mM and CYC was increased from 0 to 8.33 × 10^−6^ mM (for the sake of clarity, the red-shifts are shown with arrows).

Figure 4 shows the fluorescence spectra of (LYZ-CYC) at λ_ex_ = 280 nm and 295 nm (pH = 7.4) where the maximum fluorescence peaks occurred at 343 and 340 nm, respectively. When various concentrations of CYC were added to LYZ solution, the emission intensity dropped regularly with increasing CYC concentration and the peak position was shifted to 340–342 nm. The fluorescence spectra of LYZ at 340 nm originated from Trp62 and Trp108, that play important roles. The reduction of fluorescence intensity indicated that the CYC quenched the fluorescence of LYZ revealing that an interaction between CYC and LYZ occurred.

This interaction induced the non fluorescent complex CYC-LYZ to form. In fact, CYC affected the polarity of the micro-environment of the Tyr and Trp residues. Moreover, the red shifts suggested that micro-environment around the Trp moieties changed and became more hydrophilic because of the interaction of CYC with LYZ [1].

### 2.3. Fluorescence Quenching of LYZ-CYC Complex in the Presence of CDs (α,β,γ)

It is well known that any process that causes a decrease in the fluorescence emission can be considered as “quenching”. There are many molecular interactions that can result in such quenching, including excited-state collisional quenching, resonance energy transfer and ground-state compound formation [15]. It is known that there are two quenching mechanisms involved in quenching processes, which are usually classified as dynamic and static quenching. Static quenching is due to the formation of a ground state complex between the fluorophore and the quencher, whereas dynamic quenching results from the collision between the fluorophore and the quencher. In general, dynamic and static quenching can be distinguished by their different dependence on temperature and viscosity. The fluorescence quenching data were analyzed by the well known Stern-Volmer equation [16]:

F_0_/F = 1 + k_q_τ_0_ [*Q*] = 1 + *K*_sv_[*Q*] (1)

In Equation (1), *F*_0_ and *F* are the fluorescence intensity before and after quencher addition, respectively, *K*_sv_ is the Stern-Volmer dynamic quenching constant, a direct measure of the quenching efficiency, and k_q_ is the quenching rate constant of a biomolecule, τ_0_ and [*Q*] are the average lifetime of the biomolecule and concentration of quencher, respectively [16]. Obviously, the value of k_q_ is deduced as follows:

*K*_sv_ = k_q_τ_0_ (2)

where the term *τ*_0_ is 10^−8^ s and *K*_sv_ is the slope of the linear regressions of the *F*_0_/*F* versus [*Q*] curve. From k_q_ and *K*_sv_, the mechanism of fluorescence quenching can be clarified. The maximum scatter collision quenching constant k_q_ of various quenchers with the biopolymer (k_q.s_) is 2 × 10^10^ mol^−1^ s^−1^, if k_q_ *>* k_q.s_, it is certain that the fluorescence quenching of the biopolymer did not originate from dynamic quenching [15]. As shown in Table 1, the k_q_ value of LYZ quenching by CYC in the presence of CDs (α,β,γ) at 280 nm, is greater than k_q_, suggesting that the binding of CYC and CDs to LYZ is quite strong and that the quenching process involved a static quenching mechanism. This situation was observed during LYZ quenching in the binary and ternary systems and defined a static quenching mechanism. This means that the quenching was not initiated by a dynamic collisions but rather by the formation of a drug and CDs complex in the binary and ternary systems.

The fluorescence data was further examined using a modified Stern-Volmer equation:

F_0_/(F_0_ − F) = 1/ƒ_a_ +1/k_a_ ƒ_a_ [Q] (3)

where F_0_ and F are the fluorescence intensities before and after the addition of the quencher, respectively. k_a_ is the effective quenching constant for the accessible fluorophores, and ƒ_a_ is the fraction of accessible fluorescence. The modified Stern-Volmer plot characterizes the behavior of drug molecules when interacting with a fluorophore. When accepting the energy from excited fluorophores, drug molecules move and make space for other ligand molecules [17]. Table 1 lists the ƒ_a_ and k_q_ values for the studied binary and ternary systems at 280 nm. As can be seen, the presence of the drugs affects in ƒ_a_ the same way as reported elsewhere. When ƒ_a_ = 1, all the Trp residues are accessible to the quencher. Consequently, a change in the value of ƒ_a_ indicates that the fraction of fluorescent components accessible to the quencher was altered [18]. Table 1 indicates that ƒ_a_ = 1.2 about 80% of the total fluorescence of LYZ is accessible to the quencher [19].

**Figure 5:**
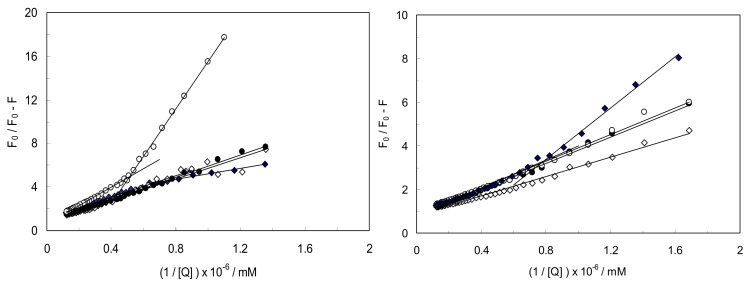
Modified Stern-Volmer plots for the fluorescence quenching of LYZ-CYC(♦), [LYZ-CD(α)]CYC(◊), [LYZ-CD(β)]CYC(●), [LYZ-CD(γ)]CYC(○); λ_ex_ = 280 nm (left; LYZ-CYC(♦), [LYZ-CD(α)]CYC(◊), [LYZ-CD(β)]CYC(●), [LYZ-CD(γ)]CYC(○); λ_ex_ = 295 nm) ([LYZ] = 1 × 10^−3^ mM, [CYC] = 5 × 10^−5^mM, [CDs] = 1 mM, pH = 7.4, T = 298 K).

Figure 5 shows the modified Stern-Volmer plots for LYZ in the presence of CDs (α,β,γ) in binary and ternary systems at excitation wavelengths of 280 nm and 295 nm. It can be seen that LYZ had two different binding sites with different affinity in binary systems. In ternary systems, CDs (α,β,γ) caused the conformational changes of LYZ and shown one binding site for CYC.

The K_sv_, k_q_ and quenching fraction of LYZ in the binary and ternary systems at λ_ex_ = 280 nm are summarized in Table 1. As can be seen from K_sv_ values in Table 1, the interaction between CYC and LYZ enhance in the presence of CDs because K_sv_ values increase, on the other hand, the kind of interaction between CYC and LYZ was different in the presence of CDs because two sets of binding sites in LYZ-CYC were altered to one set in the presence of CDs. Figure 5 shows the modified Stern-Volmer plots at an excitation wavelength of 295 nm which indicated that LYZ had two different binding constants in its interaction with CYC. The K_sv_ values of the LYZ-CYC complex at 295 nm were K_sv__1_ = 2.3 × 10^12^ M^−1^ and K_sv__2_ = 1.1 × 10^12^ M^−1^ and in the presence of CDs(α,β) they were K_sv_ = 2.3 × 10^12^ M^−1^ and K_sv_ = 2.1 × 10^13^ M^−1^, respectively. On the other hand, in the presence of CD(γ), LYZ had two different binding sites for CYC, that were K_sv__1_ = 0.7 × 10^12^ M^−1^ and K_sv__2_ = 2.9 × 10^12^ M^−1^. These values show that the various kind of CDs have different behavior in LYZ-CYC complex formation and therefore play different roles in drug delivery systems. On the other hand, the K_sv_ values of LYZ-CYC in the absence and presence of CDs at λ_ex_ = 280 nm (Table 1) and λ_ex_ = 295 nm were different, indicating that Tyr residues play important roles in LYZ-CYC complex formation in the presence of CDs.

The results suggest that the participation of Tyr and Trp groups in LYZ-CYC and CDs(α,β,γ) complexes was assessed using different excitation wavelengths. At 280 nm, the Trp and Tyr residues in LYZ were excited, whereas the 295 nm wavelength only the Trp residues are excited. A comparison of the fluorescence quenching of protein excited at 280 nm and 295 nm made it possible to estimate the participation of Trp and Tyr groups in the complex and we can see the role of Tyr in ternary systems in presence of CD (γ). In (LYZ-γ CD) CYC complex at λ_ex_ = 280 nm and λ_ex_ = 295 nm, there are one set and two sets of binding sites with one and two K_sv_ values, respectively, clearly indicating that Tyr residues play roles in complex formation.

For the static quenching interaction, if it is assumed that there are similar and independent binding sites in the biomacromolecule, the binding constant (K_a_) and the number of the binding sites (n) can be obtained from the double logarithm regression curve [Equation (4)]:

log F_0_ − F/F = log K_A_ + n log[Q] (4)

Here, K_a_ and n are the association constant and the number of binding sites, respectively [20]. The values of n_1_ and n_2_ for CYC in all systems were calculated and are listed in Table 1. According to the above equation the values of n were approximately equal to 1, which indicated that there was one set of binding sites in LYZ for CYC. The intrinsic fluorescence of LYZ primarily originates from Trp 62 and 108, and Trp 62 is more exposed to the polarity micro-environment. From the value of n, it may be speculated that CYC most likely binds to the Trp 62 and quenches its intrinsic fluorescence. It can be seen in ternary system where we had more quenched intrinsic fluorescence [21].

Measurements of the fluorescence emission and red edge excitation shift (REES) of LYZ upon interaction with CYC and CDs(α,β,γ) as binary and ternary systems made it possible to compare the environmental and mobility features of the Trp residue in the LYZ-drug complexes [22]. Red edge excitation shift (REES) is a shift in the emission maximum toward a higher wavelength caused by a shift in the excitation wavelength toward the red edge of the absorption band. The REES is due to the electronic coupling between Trp indole rings and neighboring dipoles and occurs when there are slow relaxations in solvent media. Thus, REES is particularly useful in monitoring motions around the Trp residues in protein studies [23]. For the present experiments, the choice was made to excite the Trp at both 295 nm and 305 nm to investigate the REES effect, and the results are listed in Table 2. The value of Δλ_em,max_ was defined as the difference in emission maximum between that excited at 295 nm and the one excited at 305 nm [22].

The native LYZ showed a 4 nm REES; indicating that Trp residues in the LYZ were in a slightly motionally restricted environment. In the presence of CYC; The REES values of LYZ-CYC complex was 3 nm and in ternary system. After the addition of CDs(α,β,γ); the REES value were 3 nm; 1 nm and 1 nm respectively. Decrease of Δλ_em__,__max_ meant that the introduction of CYC had an obvious lower impact on the mobility of the Trp micro-environment and that Trp residues experience less restrictions from their surroundings in the binary and ternary systems as compared to native LYZ. In other words; in the presence of CYC and CDs(α,β,γ) the values of REES decreased and the micro-environment of the Trp residues was less congested [23].

### 2.4. Synchronous Fluorescence Spectra Measurment 

Synchronous fluorescence spectroscopy technique is successfully applied to explore the micro- environment of amino acid residues by measuring the emission. It offers sensitivity, spectral bandwidth reduction, spectral simplification, and avoids different perturbing effects [24]. The shape and intensity of synchronous fluorescence spectra depend on Δλ, the difference between excitation and emission wavelength [24]. To further investigate the structural change of LYZ upon the addition of CYC, we measured synchronous fluorescence spectroscopy of LYZ with various concentrations of CYC, which is a method to study the micro-environment of Trp and Tyr residues, When Δλ is 15 and 60 nm, the synchronous fluorescence spectra of LYZ will give the environment in the vicinity of Tyr and Trp residues, respectively, and by measuring the possible shift in wavelength emission maximum λ_em_ the shift in position of emission maximum corresponding to the changes of the polarity around the chromophores molecule can be determined [25].

**Figure 6:**
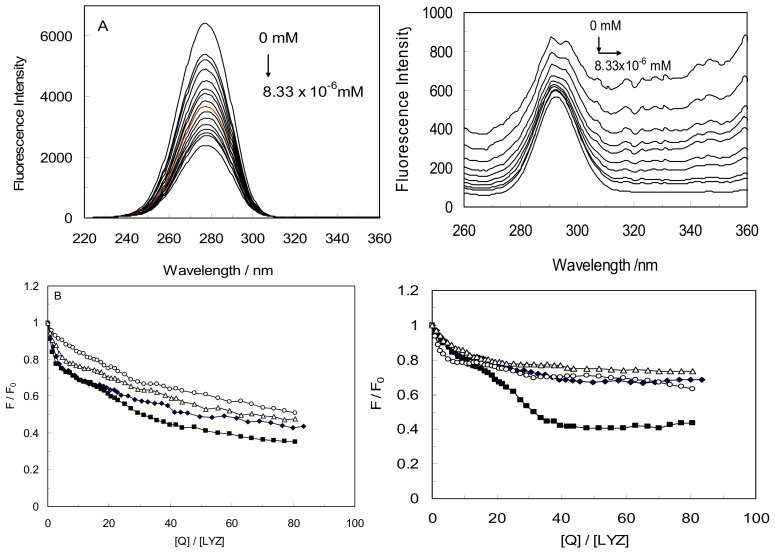
(**A**) Synchronous fluorescence spectra of LYZ in the presence of CYC, at ∆λ = 60 nm (right ∆λ = 15 nm). ([LYZ] = 1 × 10^−3^ mM, [CYC] = 5 × 10^−5^ mM, pH = 7.4, T = 298 K); (**B**) comparison of curves of F/F_0_ versus [Q] for the binary LYZ-CYC(♦), ternary [LYZ-CD(α)]CYC(■), [LYZ-CD(β)]CYC(○), [LYZ-CD(γ)]CYC(Δ), systems at ∆λ = 60 nm (right; binary LYZ-CYC(♦) system, and ternary [LYZ-CD(α)]CYC(■), [LYZ-CD(β)]CYC(○), [LYZ-CD(γ)]CYC(Δ) systems; at ∆λ = 15 nm).

The corresponding results are shown in Figure 6A. The fluorescence intensity of protein solutions decreased with increased CYC concentration. When Δλ was 60 nm, the maximum emission wavelength of the Trp residue was 276 nm for LYZ and there was an obvious shift after addition of CYC. This showed that the interaction of CYC did not affect the conformation of the Trp microregion, and the polarity in the vicinity of Trp was not changed. Figure 6A show at Δλ = 15 nm the maximum emission wavelength of the Tyr residue was 293 nm for LYZ the maximum wavelength of Tyr in the binary complex was slightly red-shifted for only 2 nm, at the investigated concentration range, shown in inset Figure 6A (right). It expressed that the conformation of Tyr microregion was changed and more hydrophilic. In other words resulting in the polarity around the Tyr residues being strengthened and the hydrophobicity weakened [24].

To explore the structural changes of LYZ-CYC in the presence of CDs(α,β,γ), we measured the curves of F/F_0_ *versus* [Q] (Figure 6B) for the LYZ-CYC system in the absence and presence of CDs at various concentrations of drug (at Δλ = 60 and Δλ = 15 nm).

By comparing the slopes of LYZ-CYC in the absence and presence of CDs(α,β,γ) at Δλ = (15,60) nm, the slope at Δλ = 60 nm was more than Δλ = 15 nm that can be deduced that the conformation of LYZ and the polarity around the Trp residues had changed and significant contribution of the Trp residues in the fluorescence of LYZ in binary and ternary systems [26]. According to the Figure 6 the slope of [LYZ-CD(α)]CYC was more than LYZ-CYC and [LYZ-CD(β,γ)]CYC when Δλ = 15 nm or 60 nm. It can be concluded for [LYZ-CD(α)]CYC that Trp and Tyr played an important role during fluorescence quenching of LYZ by comparing with other systems. Figure 6B shows the slope of the LYZ-CYC was more than that of [LYZ-CDs(β,γ)]CYC at ∆λ = 60 nm, which indicated the drug was closer to the Trp residues in the absence of CDs(β,γ) as compared to the other systems. It has been shown at ∆λ = 15 nm that the slope was similar in LYZ-CYC and [LYZ-CDs(β,γ)]CYC, indicating that the Tyr had the same role in these systems.

### 2.5. Characteristics of the Resonance Light Scattering (RLS) Spectra

Light-scattering has been widely applied to study the aggregation, size, shape and distribution of particles in solution. When the excitation wavelength is close to the absorption bands, greatly enhanced Rayleigh light-scattering signals can be expected, known as RLS [27]. In total, an enhancement of light-scattering is dependent on: (1) resonance-enhanced light scattering, (2) molecular polarizability, (3) enhancement of hydrophobicity, and (4) increase in molecular volume (or molecular weight) [28]. RLS is calculated according to the following formula:

I_RLS_ = (32π^3^V^2^n^2^ N/λ_0_^4^ )[(δn)^2^ + (δk)^2^] (5)

where n is the refractive index of the medium; N is the molarity of the solution; λ_0_ is the wavelength of incident and scattered light; V^2^ is the square of the molecular volume; and δ_n_ and δ_k_ are the fluctuations in the real and imaginary components of the refractive index of the particle, respectively [29].

When other factors are held constant, I_RLS_ is related to the square of the molecular volume, therefore with an increase in size of the formed particle and directly proportional to the molecular volume, the RLS intensity of the system becomes greatly enhanced. The RLS technique is available to provide insight into the process(es) responsible for the formation of a complex. By scanning both the excitation and emission monochromators of a common spectrofluorometer with Δλ = 0 nm, RLS spectra can be recorded [30].

**Figure 7:**
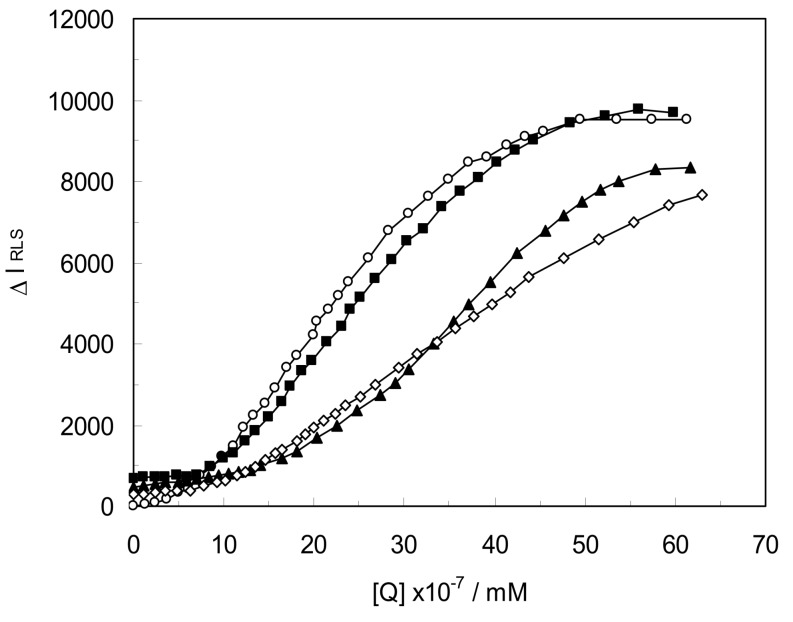
RLS spectra of LYZ in the presence concentrations of CYC in binary LYZ-CYC system(○); in ternary [LYZ-CD(α)]CYC(■), [LYZ-CD(β)]CYC(◊), [LYZ-CD(γ)]CYC(▲) systems ([LYZ] = 1 × 10^−3^ mM, [CYC] = 5 × 10^−5^ mM, [CDs] = 1 mM, pH = 7.4, T = 298 K).

Figure 7 shows the analysis of the LYZ-CYC system in the absence and presence of CDs(α,β,γ). It was found that the enhancement of the RLS intensity differed for various concentrations of the LYZ-drug solution. In the RLS spectrum, there was a non-linear relationship between the enhanced intensity and the concentration of the drug in the absence and presence of CDs(α,β,γ). When CYC and CDs concentrations were too low, the RLS intensity of the binary and ternary system hardly changed.

However, with increasing drug concentrations in all systems, the RLS intensity of the systems gradually increased, and precipitation occurred in the solutions that contained high concentrations of drugs and for a better comparison of the ability of the drugs to form complexes and aggregates on the LYZ surface. We studied the critical induced aggregation concentration (C_CIAC_) values of the interacting systems. Under identical experimental conditions, a smaller C_CIAC_ value signified a smaller concentration of drug-induced protein aggregation.

It is important to note that, smaller C_CIAC_ values testified to a higher affinity to create aggregates. Due to a greater interaction between the drug and LYZ in the presence of the CDs, the C_CIAC_ values for ternary systems were smaller than for the binary ones (the C_CIAC_ values of the LYZ-CYC was 3.7 × 10^−7^ and in [LYZ-CDs(α,β,γ)]CYC complexes were 5.9 × 10^−7^ mM, 6.3 × 10^−7^ mM, and 9.4 × 10^−7^ mM, respectively).

Consequently in the presence of CDs(α,β,γ) in the LYZ-CYC system, the affinity of CYC to form aggregates on the LYZ surface increased in the presence of CDs(α,β,γ) and the aggregated forms were generated at lower concentrations. This result showed that in ternary systems the affinity of CDs was related to the size of CDs and with enhancement size of CDs, the affinity of CDs were increased too. It can be seen the [LYZ-CD(γ)]-CYC had the maximum affinity for aggregation on LYZ.

### 2.6. Polarizability

The other possible reason for the RLS enhancement was an effect of polarizability on the scattering intensity. The light scattering formula derived by Stanton is written as follows:

I = (16 π^2^ P_N_ I_0_/λ^4^ r^2^) | ᾱ |^2^ (6)

where P_N_ is the number density of the molecules, I_0_ is the intensity of incident light, λ is the incident wavelength, r is the distance from the molecule to the observer, and ᾱ is the molecular polarizability (composed of a real and an imaginary part) [31]. It can be seen that the scattering intensity is directly proportional to the polarizability. A large increase in polarizability is thus one of the important factors for the enhancement of RLS and the formation of complexes. By means of the density function theory (DFT), the structure of LYZ and CYC was optimized [30]. At the B3LYP/LANL2MB theoretic level, the mean polarizability and energies of LYZ and CYC in the presence and absence of CDs(α,β,γ) were calculated and the values are listed in Table 3.

Our results showed that increases in mean polarizabilities of the reactants (CYC and CDs) in the binary and ternary systems. The mean polarizability of LYZ increased from 319.41 to 353.22 and to 347.73, 347.02, 346.11 in binary and ternary systems, respectively. The extent of reaction is significant, providing evidence for the enhancement of RLS. On the other hand, the polarizabilities of the complex in the binary system increased. It was observed that the polarizabilities of the binary systems were more than for ternary counterparts.

This is the reason for the increase in RLS intensity during complex formation. In other words, the presence of the CDs(α,β,γ) caused a reduction of the polarizability values in the ternary system that show the complex formation between LYZ and CYC in the presence of CDs(α,β,γ).

### 2.7. Energy Transfer Efficiency and Binding Distance

**Figure 8:**
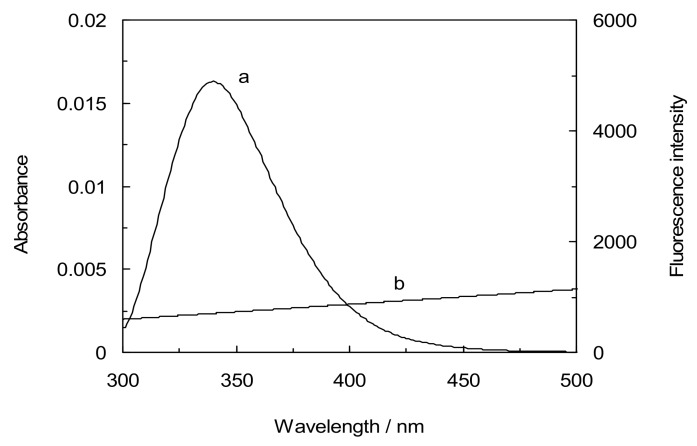
Spectral overlap of the fluorescence emission spectrum (curve a) of LYZ with the absorption spectrum (curve b), [LYZ] = 1 × 10^−3^ mM, [CYC] = 5 × 10^−5^ mM, [CDs] = 1 mM, pH = 7.4, T = 298 K.

Fluorescence resonance energy transfer (FRET) is a distance dependent interaction between the different electronic excited states of molecules in which excitation energy is transferred from one molecule (donor) to another molecule (acceptor) without emission of a photon from the former molecular system. According to Förster’s theory, the efficiency of FRET depends mainly on the following factors: (i) the extent of overlap between the donor emission and the acceptor absorption; (ii) the orientation of the transition dipole of donor and acceptor, and (iii) the distance between the donor and the acceptor. Here the donor and acceptor are LYZ and CYC, respectively. There was a spectral overlap between the fluorescence emission spectrum of lysozyme and UV/vis absorption spectrum of CYC (Figure 8) [32]. According to Förster’s non-radiative energy transfer theory, the distance (r) between the donor (LYZ) and the acceptor (CYC) can be calculated by equations:

E = R_0_^6^/R_0_^6^+ r^6^ =1 − F/F_0_ (7)

R_0_ = 8.8 × 10^−25^K^2^n^−4^φJ (8)

where F and F_0_ are the fluorescence intensities of biomolecule in the presence and absence of quencher, r the donor–acceptor distance and R_0_ the critical distance where the transfer efficiency is 50%, K^2^ the spatial orientation factor of the dipole, n the refractive index of the medium, φ the fluorescence quantum yield of the donor, and J the overlap integral of the fluorescence emission spectrum of the donor and the absorption spectrum of the acceptor [33]:

J = (F (λ) ε (λ_0_) λ^4^ Δλ)/(F (λ) Δλ ) (9)

where J is the effect of the spectral overlap between the emission spectrum of the donor and the absorption spectrum of the acceptor, F(λ) the corrected fluorescence intensity of the donor in the wavelength range λ_0_ to λ, and ε(λ_0_) the extinction coefficient of the acceptor at λ_0_. In present study the value of J, E, R_0_ (nm), r listed in Table 4 [34].

The overlap of the fluorescence emission spectra of LYZ with the UV absorption spectra for CYC in the binary systems are shown in Figure 8. The distance between CYC and flurophore in LYZ was 2.67 nm in the absence of CDs(α,β,γ), whereas it was 2.77 nm, 2.83 nm and 2.88 nm for amino acids in LYZ in the presence of CDs(α,β,γ), respectively, as can be seen in Table 4. It can furthermore be observed that these values increased with presence of CDs and that all of them were lower than 7 nm in the interaction betwen LYZ and CYC with absence and presence of CDs.

This is in accord with the conditions of Förster’s non-radiative energy transfer theory. Moreover, these results suggest a static quenching mechanism in the interaction between the drug and LYZ in the absence and presence of CDs. The results illustrated that, in the presence of CDs(α,β,γ), in ternary systems the distance between the drug and LYZ increased, indicating that CDs caused a decrease in the energy transfer of LYZ to CYC.

### 2.8. Circular Dichroism (CD) Analysis

CD is a vigorous analytical technique to investigate the association of proteins with other ligands and to determine the protein conformation in solution. The CD curves of LYZ denoted two negative peaks in the far-UV CD region at 208 nm and 222 nm, typical of a protein α-helical structure, both contributed to by *n*→π* transfer for the peptide bond transfer for the peptide bond of the *α*-helix. This reflects the largely helical conformation of the protein, and if the *α*-helix changes, the spectra will change accordingly [18,35].

**Figure 9:**
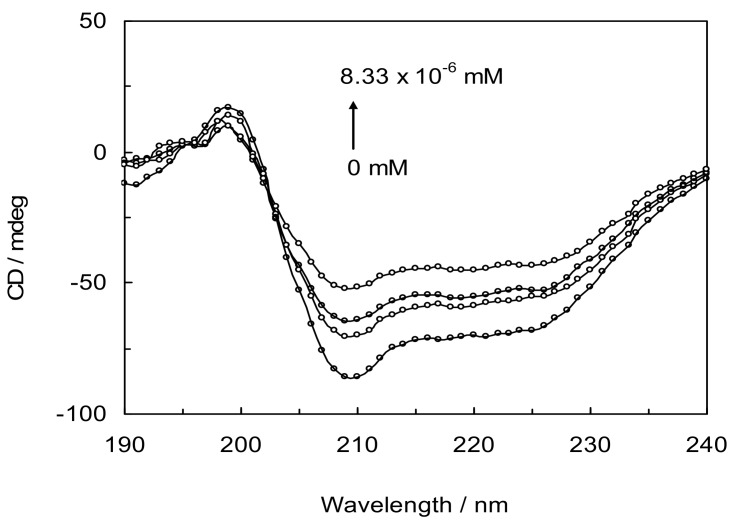
Far-UV CD spectra of LYZ and CYC. pH = 7.4 and T = 298 K. [LYZ] = 1 × 10^−3^ mM, The concentrations of CYC ranged from 0 to 8.33 × 10^−6^ mM.

The two characteristic peaks were found to decrease with increasing drug concentrations. The data were expressed as the molar residue ellipticity [*θ*], defined as [*θ*] = 100*(θ*_obs_*/cl)*, where *θ*_obs_ is the observed ellipticity in degrees, *c* is the concentration in mol residue cm^−3^, and *l* is the length of the light path in cm. To analyze the structural changes of LYZ quantitatively, the raw CD spectra of LYZ with CYC in the absence and presence of CDs were scanned (Figure 9) and secondary structure components computed based on CD data are listed in Table 5 [36,37].

The results show that *α*-helical content (regular and distorted) and β-sheet (regular and distorted) decrease and the content of unordered coil increases after addition of drug and CDs(α,β,γ) respectively. A decrease in *α*-helical content and an increase in unordered coil structures were observed with the adsorbed LYZ. CDs have hydrophobic core that can be interacted with hydrophobic residues of LYZ and destabilize it, therefore the secondary structure of LYZ in the presence of CDs deceases as shown in Table 5.

The results suggest that the LYZ molecules probably adopt a looser conformation with the extended polypeptide structures that show the hydrogen bond alteration in the LYZ. The conformational transition probably results in the exposure of the hydrophobic cavities and a perturbation of micro-environment surrounding the deprotonated aromatic amino acid residues, which are favourable for the LYZ adsorption on to the surface of CYC and CDs. Furthermore, it can be concluded that the occupancy of the Trp sites by the binding ligands could actually destabilize the native conformation of the protein.

### 2.9. Molecular Modeling

Molecular modeling studies were performed on the basis of the relationship between the docking energy of drug-protein complexes and it was calculated as the sum of the Van Der Waals and electrostatic interaction energies [38]. As binding of ligands to protein affects the distribution and intensity of its pharmacological and toxicological action, we undertook a molecular modeling and docking study to explore the specific binding region of CYC and CDs to LYZ [39].

LYZ is a small monomeric protein constituted by 129 amino acids folded in two domains, one is described by four α-helical structures encompassing the amino- and carboxy-terminal segments, and a triple-stranded antiparallel β-sheet that, together with a long loop, makes up much of the second domain [19,40]. Both domains are functional for the active site cleft which is formed between them, and stabilized by four disulfide bonds (6↔127, 30↔115, 64↔80, and 76↔94). LYZ includes six Trp residues at positions 28, 62, 63, 108, 111, and 123, two of which (Trp-62 and Trp-108) are responsible for most of its intrinsic fluorescence, and for the others in the proximity of the Trp with the sulfur atoms of the disulfide bonds [19].

**Figure 10:**
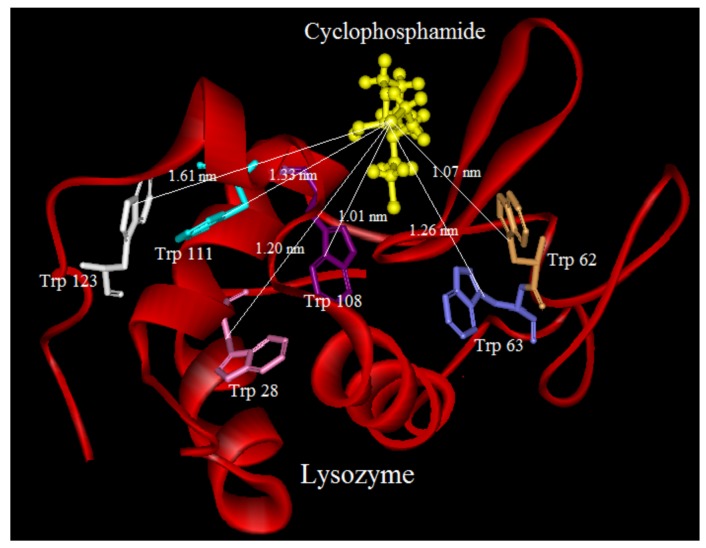

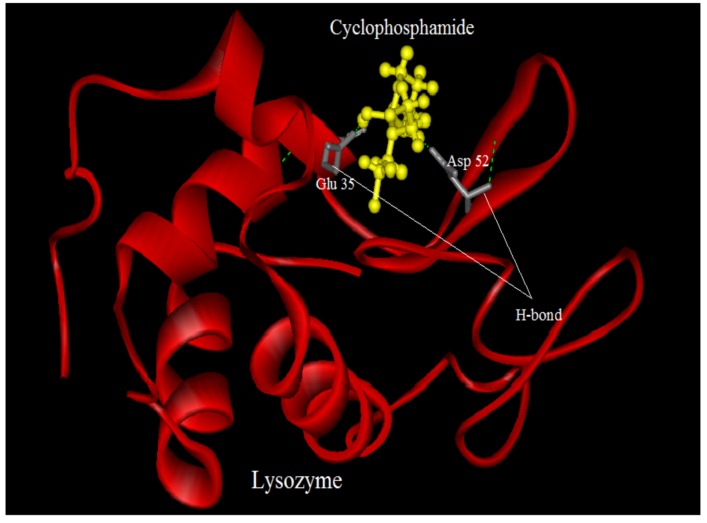
Distance between CYC and Trp residues in binary system and in the absence of CDs; below: show the number and type of amino acids involved in the hydrogen binding between LYZ and CYC in absence of CDs.

**Figure 11:**
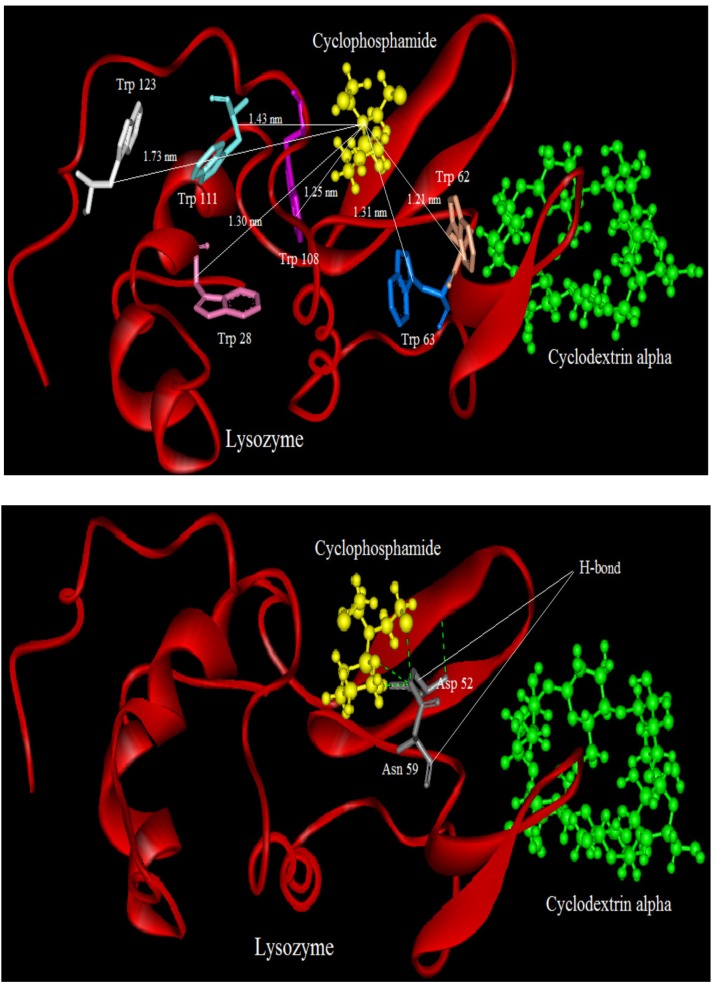
Distance between CYC and Trp residues in ternary system and in the presence of CD(α); below: show the number and type of amino acids involved in the hydrogen binding between LYZ and CYC in presence o of CD(α).

**Figure 12:**
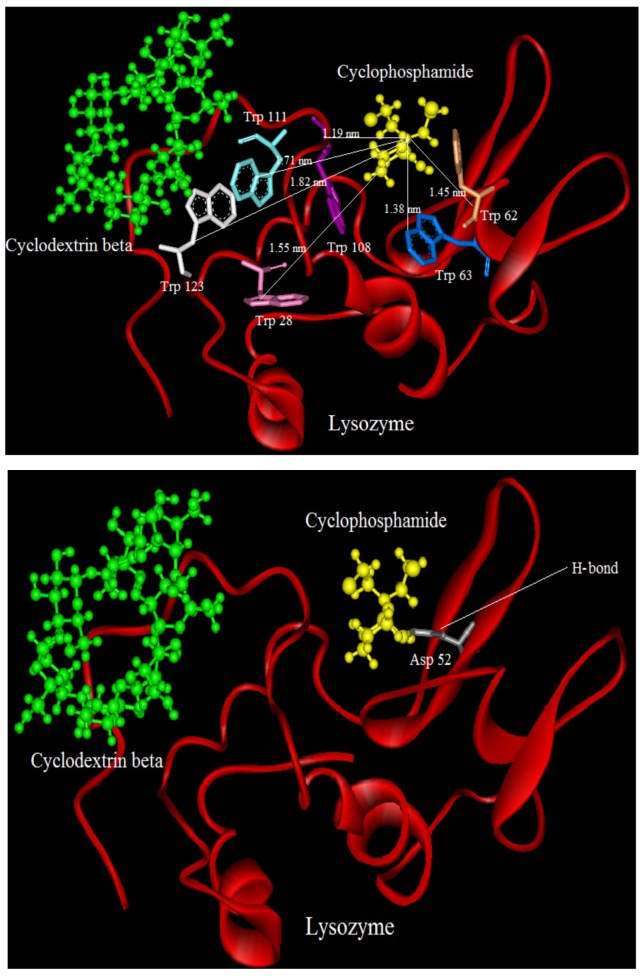
Distance between CYC and Trp residues in ternary system and in the presence of CD(β); below: show the number and type of amino acids involved in the hydrogen binding between LYZ and CYC in presence o of CD (β).

**Figure 13:**
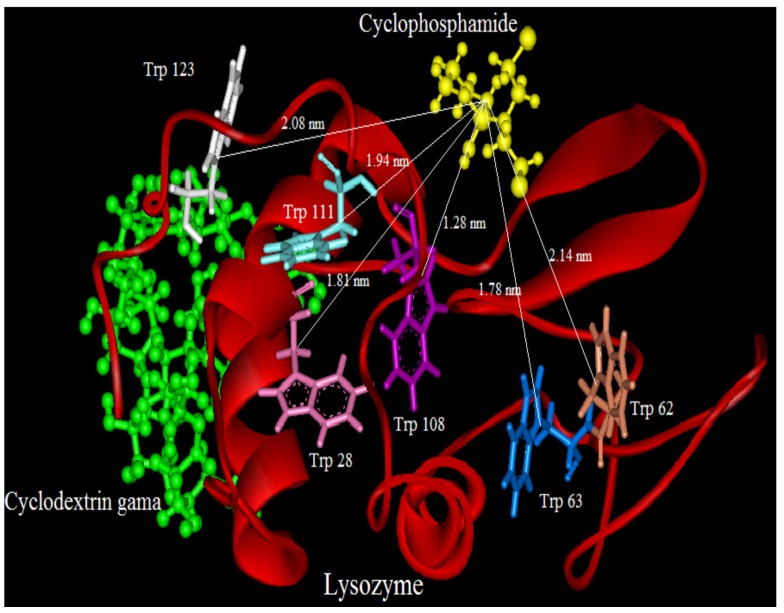

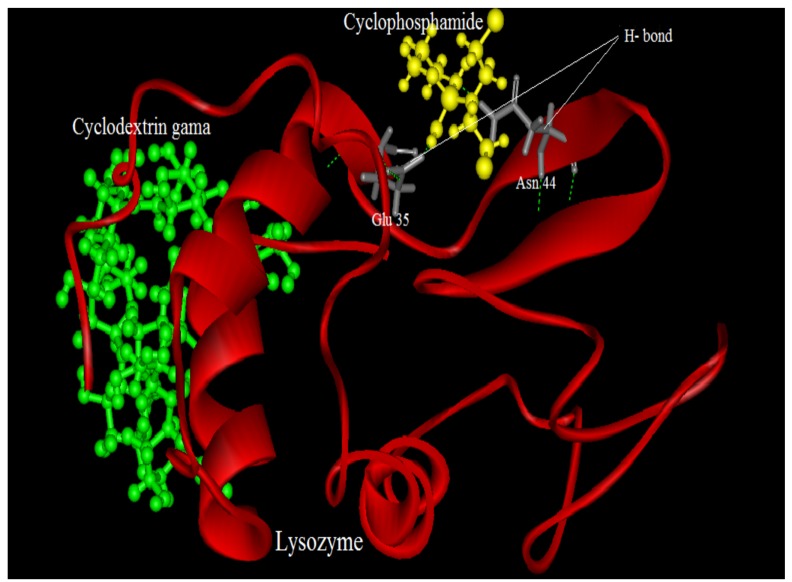
Distance between CYC and Trp residues in ternary system and in the presence of CD(γ); below: show the number and type of amino acids involved in the hydrogen binding between LYZ and CYC in presence o of CD(γ).

We tried to estimate possible localization sites “binding sites” of CYC in presence and absence of CDs(α,β,γ) at the proteins surface. The crystal structure of LYZ (PDB entry 6LYZ) was downloaded from the Protein Data Bank and used for the docking studies. The best docking result of interaction between CYC and LYZ in the absence and presence of CDs is shown in Figure 10, Figure 11, Figure 12 and Figure 13.

It can be observed that LYZ has different modes of interaction in the absence and presence of CDs. As seen in Figure 10, Figure 11, Figure 12 and Figure 13, the molecular distance between drug and Trp residues (28, 62, 63, 108, 111, 123) was measured in the absence and presence of CDs (α,β,γ).

In binary systems the average distance between drug and Trp residues was 1.24 nm and in the presence of CDs (α,β,γ) the average distances were 1.37 nm, 1.51 nm, 1.83 nm, respectively. These findings provided a good structural basis to explain how the very efficient fluorescence quenching of Trp residues its emission in the presence of CYC and CDs in all interacting systems.

The results show that in ternary systems the average distances between drug and Trp residues were more than that in binary systems, while the energy transfer of LYZ to CYC is decreased in ternary systems the distances between CDs and Trp residues increase. The insets of Figure 10, Figure 11, Figure 12 and Figure 13 show the number and type of amino acids involved in the hydrogen binding between LYZ and CYC in the presence and absence of CDs. In binary systems there were two hydrogen bonds with the amino acids Asp 52 and Glu 35. The ternary system in the presence of CD(α) formed two hydrogen bonds with the amino acids Asp 52 and Asn 59, while CYC in the presence of CD(β) was able to form a hydrogen bond with Asp 52 and in the presence of CD(γ) it formed a hydrogen bond with Asn 44 and Glu 35, resulting in more stabilization in its pocket. The K_i_ values obtained from molecular modeling are not truly the same values of K_SV_ but the pathway changes in molecular modeling and fluorescence spectroscopy are the same. The results obtained from molecular modeling confirmed the data from our previous experiments, such as the synchronous fluorescence and far-UV CD analysis, and were in accordance with the Stern-Volmer binding affinity and other results.

## 3. Experimental 

### 3.1. Materials and Solutions

Hen egg white LYZ, CYC and CDs (α,β,γ) were purchased from the Sigma Chemical Corporation (New York, NY, USA). The protein was dissolved in 50 mM phosphate buffer solutions at pH 7.4 and was prepared with the following concentrations: [LYZ] = 1 × 10^−3^ mM under physiological conditions. The CYC solution (5 × 10^–5^ mM) and the CD solutions (1 mM) were prepared by dissolution in a phosphate buffer (pH 7.4). All solutions were made up at room temperature and were stored in a refrigerator at 4 °C in the dark.

### 3.2. Apparatus

All fluorescence spectra were recorded on an F-2500 fluorescence spectrophotometer (Hitachi, Tokyo, Japan) equipped with a xenon lamp light source, and 1.0-cm quartz cells were used for the measurements. The excitation wavelength was set to 280 nm and 295 nm and 305 nm, and the emission wavelength was recorded between 300 nm and 500 nm and the excitation and emission slit widths were set to 5 nm. The scan speed was 1200 nm/min, and the Photo Multiplier Tube (PMT) voltage was 700 V. Fluorescence intensities were corrected for inner filter and dilution effects before analysis of the binding and quenching data. All the experiments were repeated at least three times and performed at room temperature.

Absorbance measurements were carried out with a Jasco spectrophotometer (V-630 model, Tokyo, Japan) equipped with a personal computer and 1.0-cm quartz cells. The optical system was based on a split-beam with a grating bandwidth of 5 nm, and the light source was a xenon lamp and the wavelength range was 200–500 nm.

Resonance light scattering (RLS) spectra were recorded by scanning both the excitation and emission monochromators of a common spectrofluorometer with ∆λ = 0 nm. All RLS spectra were obtained by simultaneously scanning the excitation and emission monochromators (namely ∆λ = 0 nm) from 220 nm to 600 nm with slit widths of 5 nm for the excitation and emission.

Synchronous fluorescence spectroscopy was carried out by simultaneousely scanning the excitation and emission monochromators. Far-UV CD experiments were performed on a Jasco-815 spectropolarimeter equipped with a Jasco 2-syringe titration mechanism. Spectra were recorded with the same protein concentration in a 1-mm path length quartz cuvette. A bandwidth of 1 nm was used together with a response time of 2 s, with a scanning rate at 50 nm·min^−1^ to obtain the final spectrum as an average of three scans. The instruments were calibrated with ammonium d-10-camphorsulfonic acid. The induced ellipticity, given in degrees, was obtained by the ellipticity of the drug-protein mixture after subtraction of the ellipticity of the drug at the same wavelength. All pH measurements were performed with a Metrohm digital pH-meter (Metrohm, Berlin, Germany).

Resonance energy transfer measurements experiments were obtained the binding distance (r) between a protein (donor) and a bound drug molecule (acceptor), along with the relative angular orientation of fluorophores, can be calculated from Förster’s theory, as the donor (the excited fluorophore) and acceptor (a chromophore or a fluorophore) can be entirely separate or attached to the same macromolecule.

A Jasco 815 double-beam spectrophotometer linked to a personal computer was used to record the absorption spectra of the CYC in the range of 200–700 nm at room temperature in quartz cells with 1-cm path lengths. The system was first base lined with buffer solution. Then, the overlap of the UV absorption spectrum of CYC with the fluorescence emission spectrum of LYZ and LYZ-CDs was used to calculate the energy transfer. For the energy transfer experiments, the excitation wavelengths were fixed at 295 nm.

### 3.3. Molecular Modeling

The crystal structure of LYZ was retrieved from RCSB Protein Data Bank (PDB entry: 6LYZ). MOE was used as the tool to prepare the final ligand, docking procedure, analysis of the active site of a protein-ligand complex with known structure, docking of a new ligand to the protein and analysis of the docked complexes. By employing MOE tools in the docking procedure we assumed drugs as flexible molecules and let the docking software rotate all rotable bonds of the drug to determine the best and optimized conformations of the drug within the active site of the protein.

The drug structure was built in the MOE environment (Builder) and the energy was minimized while the structure of the CDs (α,β,γ) was built by ChemBioDraw and ChemBio3D followed by saving the corresponding Mol file which was then used in the Hyperchem7 program for final energy minimization with the semi-empirical AM1 method and RMS equal to 0.0.1 Kcal/mol. We also utilized the ViewerLite, SPDBV and Molegro programs for deeper analysis of the docking results and recording the images of the study.

### 3.4. Procedures

LYZ and CYC were dissolved in a phosphate buffer, at concentrations of 1 × 10^−3^ mM and 5.0 × 10^−^^5^ mM, respectively. CYC concentration was below the common usage doses, which was varied. To a 1.0-cm quartz cell, LYZ solution added in order to make up 2 mL, and the range of the drug CYC solution was gradually titrated manually into the cell using a micro-injector for the binary systems. The fluorescence spectra were measured (with an excitation wavelength at 280 nm and 295 nm, and an emission wavelength of 300–600 nm). The entrance and exit slit widths were 5 nm and the scanning speed was 240 nm·min^–1^, making it possible to obtain both fluorescence quenching spectra and synchronous fluorescence spectra only showing the Tyr and Trp residues of LYZ when the wavelength interval (Δλ) was 15 nm and 60 nm, respectively. For the ternary systems the concentrations of cyclodexterins was 1 mM. The interaction time was investigation and the results showed that 3 min was enough for stabilization.

The UV absorbance spectra of CYC in the presence and absence of cyclodexterins were recorded and spectral scanning curves were created under the same conditions. For RLS measurement the excitation and emission monochromators were scanned simultaneously in the presence and absence of cyclodextrins with a constant wavelength interval of Δλ = 0 (λ_em_ = λ_ex_) from 220 nm to 800 nm. For exploring changes in the secondary structure the far-UV CD spectra were obtained over a wavelength range of 190–240 nm in the absence and presence of cyclodextrins under the same condition as described above. In all titration experiments, the dilution factor of ligand titration was corrected.

## 4. Conclusions

In this paper, the interactions of CYC with LYZ in the absence and presence of CDs were studied by measuring the fluorescence, synchronous fluorescence, UV-Vis absorption spectroscopy and molecular modeling approaches. The quenching fluorescence mechanism of LYZ by CYC was a static quenching. The main forces were hydrophobic and electrostatic forces. The change in the micro-environment around the Trp and Tyr residues of the LYZ molecules during the binding process has been confirmed by the synchronous fluorescence measurement in binary and ternary systems. Steady state fluorescence testifies to the fact the quenching of LYZ was mostly the result of a static mechanism. Based on Förster’s nonradiative energy transfer theory (FRET), the donor–acceptor distances of CYC–LYZ and CDs were calculated and indicated that the process of CYC binding to LYZ was accompanied by energy transfer. Additionally, the secondary structure of LYZ proved to be changed after the addition of CYC and presence and absence of CDs with reduction of (regular and disordered) α-helix accompanied by an decreased (regular and disordered) β-sheet and enhancement of turn, and random- coil, symbolizing a partial destabilization of the protein. According to the docking results, the binding site of CYC to LYZ was determined in the binary and ternary systems. Our results showed that CDs can be used for drug delivery in drug binding proteins. On the other hand, the size of CDs plays an important role in the LYZ-CYC complex formation, therefore the different forms have different roles in drug delivery.

## Figures and Tables

**Table 1:** The values of Stern-Volmer quenching constants (K_sv_), number of binding sites (n), fractional of accessible protein (f) and correlation coefficient (R) in the binary and ternary systems at λ_ex_ = 280 nm.

System	K_sv1_ × 10^−12^ M^−1^	K_sv2_ × 10^−12^ M^−1^	k_q1_ × 10^−20^ M^−1^s^−1^	k_q2_ × 10^−20^ M^−1^s^−1^	n_1_	n_2_	f_1_	f_2_	R_1_	R_2_
LYZ-CYC	2.4 ± 0.01	2.2 ± 0.02	2.4 ± 0.02	2.2 ± 0.02	1.21	1.55	1.23	0.74	0.99	0.99
(LYZ-α CD)CYC	3.6 ± 0.02	−−	3.6 ± 0.02	−−	1.14	−−	1.28	−−	0.99	−−
(LYZ-β CD)CYC	2.5 ± 0.02	−−	2.5 ± 0.02	−−	1.16	−−	1.22	−−	0.99	−−
(LYZ-γ CD)CYC	2.7 ± 0.01	−−	2.7 ± 0.01	−−	1.02	−−	1.25	−−	0.99	−−

**Table 2:** Red edge excitation shift (REES) effects for LYZ and binary and ternary LYZ-drug complexes at λ_ex_ = 305 nm and λ_ex_ = 295 nm, pH 7.4, 298 K.

Sample	λ_em__,max_/nm		REES value (nm)
	λ_ex_: 295 nm	λ_ex_: 305 nm	
LYZ	339	343	4
LYZ-CYC	340	343	3
(LYZ- α CD)CYC	340	343	3
(LYZ- β CD)CYC	342	343	1
(LYZ- γ CD)CYC	342	343	1

**Table 3:** Calculated effect of polarizabilities and energies of LYZ with CYC in the presence of cyclodextrins.

System	Polarizability/a.u
LYZ CYC LYZ - CYC α CD β CD γ CD (LYZ- α CD) CYC (LYZ- β CD) CYC (LYZ- γ CD) CYC	319.41 50.06 353.22 39.03 42.25 43.76 347.73 347.02 346.11

**Table 4:** The distance, r, between donor and acceptor of LYZ with CYC and cyclo- dexterins as binary and ternary systems (pH = 7.4, T = 298 K).

System	J (cm^3^ L·mol^−1^)	E	R_0_ (nm)	r (nm)
LYZ-CYC (LYZ- α CD)CYC (LYZ- β CD)CYC (LYZ- γ CD)CYC	1.21 × 10^−14^ 1.37 × 10^−14^ 1.54 × 10^−14^ 1.79 × 10^−14^	0.5 0.53 0.57 0.62	2.53 2.61 2.75 2.93	2.67 2.77 2.83 2.88

**Table 5:** Secondary structural analysis of LYZ-CYC complex in the absence of CD(α,β,γ) at binary and ternary systems. [CD(α,β,γ)] = 1 mM in all experiments.

System	H(r)%	H(d)%	S(r)%	S(d)%	Tr%	Un%
LYZ	16.82	7.45	22.13	14.06	6.17	33.37
LYZ-CYC	13.46	5.22	20.14	14.01	6.03	41.14
(LYZ- α CD)CYC	12.09	4.66	18.28	12.84	5.86	46.27
(LYZ- β CD)CYC	11.71	4.51	18.03	12.81	5.82	47.12
(LYZ- γ CD)CYC	10.31	3.17	16.24	10.13	5.61	54.54

(H(r) regular alpha-helix, H(d) disordered alpha-helix, S(r) regular beta-sheet, S(d) disordered beta-sheet, Tr Turn, Un unordered structure).
